# Intravitreal dexamethasone implant therapy for the treatment of cystoid macular Oedema due to hydroxychloroquine retinopathy: a case report and literature review

**DOI:** 10.1186/s12886-018-0985-x

**Published:** 2018-12-06

**Authors:** Seong Joon Ahn, Jooyoung Joung, Sang Hyup Lee, Byung Ro Lee

**Affiliations:** Department of Ophthalmology, Hanyang University Hospital, Hanyang University College of Medicine, #17 Haengdang-dong, Seongdong-gu, Seoul, 133-792 South Korea

**Keywords:** Cystoid macular oedema, Dexamethasone implant, Hydroxychloroquine retinopathy

## Abstract

**Background:**

Cystoid macular oedema (CMO) is an uncommon complication associated with hydroxychloroquine (HCQ) retinopathy threatening central vision. We report a patient with HCQ retinopathy and CMO, for which an intravitreal dexamethasone implant was used, which led to complete resolution of oedema.

**Case presentation:**

A 57-year-old woman with systemic lupus erythematosus (SLE) complaining of blurred vision in both eyes was diagnosed with bilateral HCQ retinopathy and CMO based on characteristic photoreceptor defects and cystoid spaces on optical coherence tomography, hypo-autofluorescence on fundus autofluorescence, and corresponding visual field defects. After treatment with systemic acetazolamide and topical dorzolamide, CMO showed partial resolution in the right eye. Owing to worsening renal function, an intravitreal dexamethasone implant was placed in the right eye, which resulted in resolution of CMO and visual improvement from 20/50 to 20/30.

**Conclusion:**

Intravitreal dexamethasone implant may be effective for the treatment of CMO in HCQ retinopathy, particularly for the cases refractory to systemic or topical carbonic anhydrase inhibitors.

## Background

Hydroxychloroquine (HCQ) is widely used for the treatment of several rheumatologic and dermatologic diseases such as rheumatoid arthritis and systemic lupus erythematosus (SLE). [1] Retinal toxicity caused by the drug, called HCQ retinopathy, is a well-known condition, characterized by photoreceptor and/or retinal pigment epithelium defects. This is irreversible and progressive in nature, and requires careful and regular screening for early detection. [1]

Cystoid macular oedema (CMO) in eyes with HCQ retinopathy has been documented in several reports. [2–7] Kellner et al. reported cases with CMO secondary to HCQ retinopathy, [2] and the beneficial effect of systemic acetazolamide and topical dorzolamide in one of three patients. However, successful results with systemic or topical carbonic anhydrase inhibitor therapy for CMO associated with HCQ retinopathy have been documented in other, recent reports. [3, 4]

A biodegradable intravitreal dexamethasone implant (Ozurdex; Allergan Inc., Irvine, CA) has shown effectiveness for resolution of macular oedema, including cystoid macular oedema, associated with other aetiologies. [8] Herein, we report a case of SLE complicated with CMO, for which intravitreal dexamethasone therapy was performed and resulted in complete resolution of CMO without any significant ocular complication. We also summarize the previous reports on the treatment of CMO in eyes with HCQ retinopathy, to highlight the therapeutic efficacy of intravitreal dexamethasone implant for this condition.

## Case presentation

**Fig. 1:**
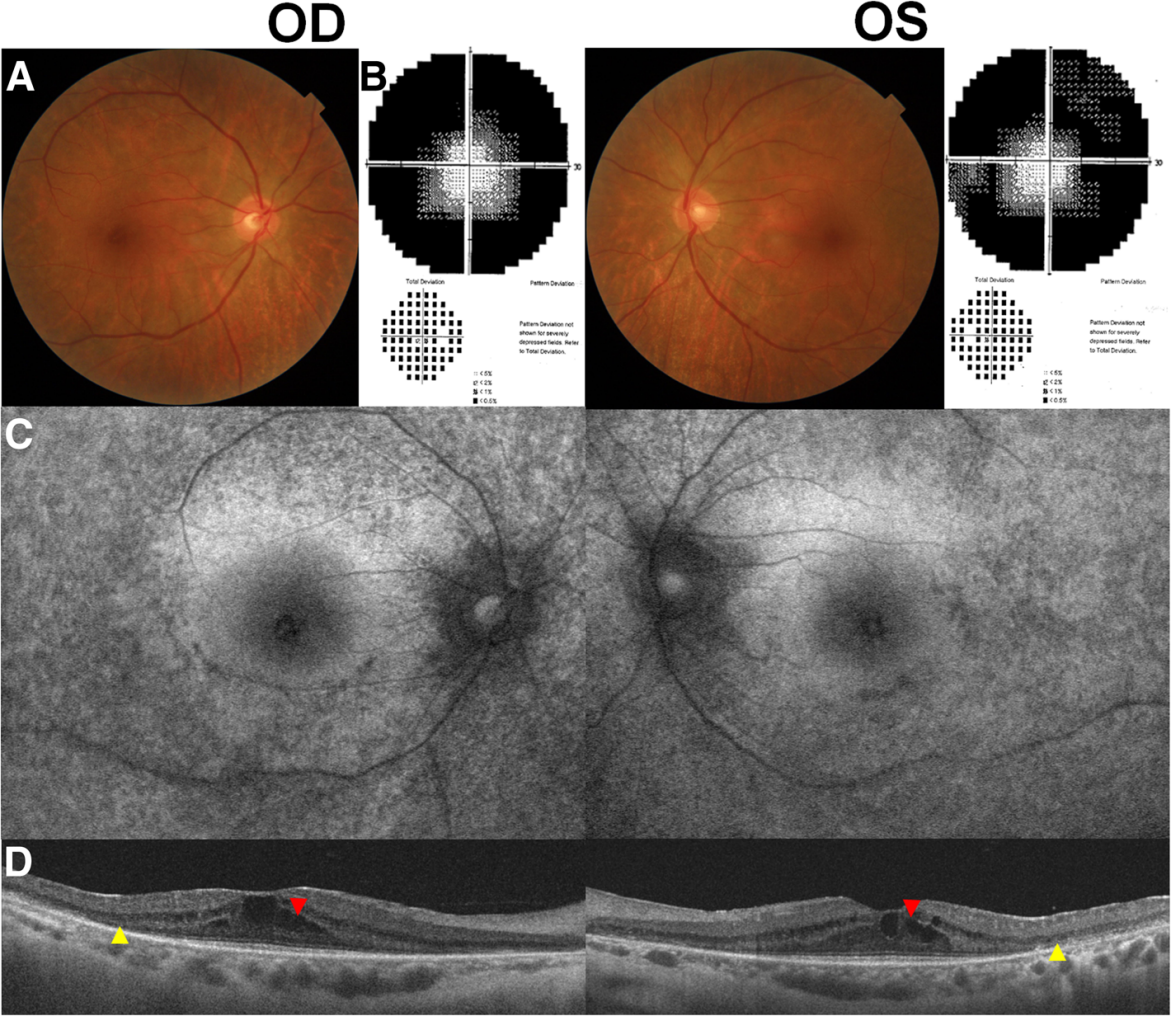
Fundus photographs (**a**), visual field results (**b**), fundus autofluorescence (FAF; **c**), and optical coherence tomography (OCT; **d**) images of the 57-year-old female patient on hydroxychloroquine therapy for 20 years. At the initial visit, mild pigmentary change on fundus photographs, photoreceptor defects on OCT (yellow arrowheads), and hypo-autofluorescence on FAF were observed in the pericentral areas. OCT also revealed cystoid spaces within the retina at baseline (red arrowheads). Humphrey 30–2 visual field examination revealed field constriction in both eyes

A 57-year-old woman visited our clinic complaining of blurred vision in both eyes that had started 4 months previously. She had been diagnosed with SLE and had been on HCQ therapy (200 mg/d) for the past 20 years. The cumulative dose of HCQ was approximately 2190 g. She did not report any history of systemic diseases that could be associated with ME, such as diabetes and hypertension. Her best-corrected visual acuity (BCVA) was 20/50 in both eyes and fundus examination showed midperipheral pigmentary changes in both eyes (Fig. 1a). Visual field (VF) examination showed field constriction on the grayscale map at baseline (Fig. 1b). Fundus autofluorescence (FAF) showed bilateral pericentral hypo-autofluorescence (Fig. 1c). Optical coherence tomography (OCT) revealed photoreceptor defects (yellow arrowheads) in the pericentral area and cystoid spaces (red arrowhead) in the macula of both eyes (Fig. 1d). Based on her medical history, characteristic photoreceptor defects on OCT, and corresponding findings on FAF and VF examination, she was diagnosed with HCQ retinopathy associated with CMO. Accordingly, HCQ treatment was discontinued by the prescribing physician.

**Fig. 2:**
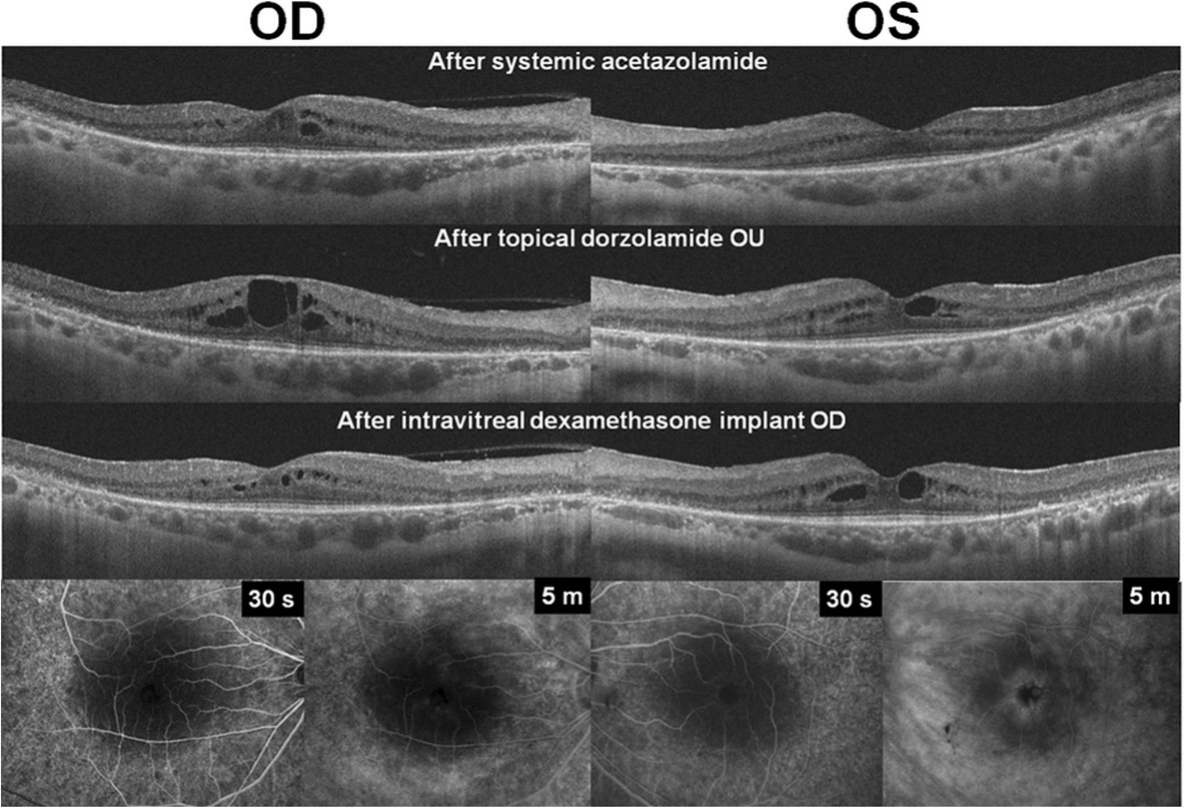
Optical coherence tomography and fluorescein angiography images obtained after the treatment of cystoid macular oedema (CMO). For the treatment, systemic acetazolamide resolved CMO in both eyes. After discontinuation of systemic acetazolamide due to deteriorating renal function, topical dorzolamide was used and resulted in aggravation of CMO. Intravitreal dexamethasone implant, used in the right eye, showed resolution of CMO at 1 month following the procedure. Fluorescein angiography images (E) obtained after the dexamethasone implant therapy revealed decreased leakage on the macula in the treated eye compared to the fellow eye

Oral acetazolamide (250 mg once a day) therapy was started. One month later, OCT revealed partial resolution of CMO in the right eye, as demonstrated by decrease in the size of cystoid spaces (Fig. 2) and in central foveal thickness (CFT) from 418 to 335 μm, and complete resolution of CMO in the left eye (CFT decreased from 338 to 255 μm). Her BCVA was 20/50 in the right eye and 20/30 in the left. However, her rheumatologist recommended discontinuation of oral acetazolamide due to the risk of aggravation of the patient’s underlying renal issue, lupus nephritis, as her renal function, as measured by glomerular filtration rate, had decreased from 70 to 56 mL/min/1.73 m^2^ during the one-month period. We discontinued systemic acetazolamide accordingly; topical dorzolamide 2%/timolol maleate 0.5% fixed combination (Cosopt; MSD Korea) twice daily was started in its stead.

Three months later, she complained of worsening of blurred vision. Her BCVA was 20/50 in both eyes, and OCT revealed aggravated CMO, as demonstrated by an increase in CFT, to 427 and 348 μm in the right and left eyes, respectively (Fig. 2). After explaining to her the possible therapeutic options, we treated her with intravitreal injection of 0.7 mg dexamethasone implant (Ozurdex) and discontinued topical dorzolamide in the right eye.

One month after the therapy, the patient reported that her blurred vision had improved; her BCVA was 20/30 in the right eye and 20/40 in the left. Fluorescein angiography (FA) showed decreased leakage in the right eye in the late phase compared to the untreated, fellow eye (Fig. 2). The OCT also revealed regression of CMO in the right eye. CFT in the right eye was decreased to 284 μm, whereas that in the left showed no remarkable change (from 348 to 347 μm). The resolution of CMO persisted up to the last visit, 2 months after the dexamethasone implant was placed. Her intraocular pressure was 15 and 11 mmHg in the right and left eye, respectively, and no other ocular complication was noted at the last visit.

## Discussion

CMO is an uncommon complication of HCQ retinopathy. As patients with HCQ retinopathy usually show parafoveal or pericentral pattern of outer retinal damage, central vision is generally preserved until the condition has progressed to an advanced stage. However, CMO can be a significant threat to vision in this patient group as it involves the central macula. However, whereas HCQ retinopathy is irreversible, appropriate treatment for CMO is possible and imperative to preserve visual function.

There have been several reports on the treatment of CMO associated with HCQ retinopathy. Table 1 summarizes the results of various CMO therapies reported in the literature. Several authors reported that cases with CMO showed beneficial response to systemic acetazolamide or topical dorzolamide. [2–4] More specifically, use of systemic and topical acetazolamide led to resolution of CME in 2 of 3 and 1 of 2 reports, respectively. The role of systemic or topical acetazolamide has been suggested to involve an enhanced pumping function of fluid mediated by the RPE, which was defective due to RPE damage in eyes with HCQ retinopathy. These results imply that systemic or topical acetazolamide may be effective and safe as the first-line treatment of CME in HCQ retinopathy. In the present case, we also observed the therapeutic effect of systemic carbonic anhydrase inhibitors for the condition; however, a long-term, continuous treatment was necessary, as the discontinuation of systemic acetazolamide led to recurrence or aggravation of CMO in the patient. Of note, SLE, one of the most common indication for HCQ therapy, may also involve the kidney and thus, long-term use of systemic carbonic anhydrase inhibitors may be detrimental to renal function. Considering such systemic risks, local treatment, i.e. intraocular injection, might be more desirable for the treatment of CMO than systemic carbonic anhydrase inhibitors, particularly in patients with associated renal diseases.

**Table 1:** Clinical efficacies of various treatment modalities for cystoid macular oedema associated with hydroxychloroquine retinopathy reported in the literature

Reference (Year)	Treatment	No. of cases	Anatomic outcome	Visual outcome	Complications
Kellner et al. (2014)	Topical dorzolamide or systemic acetazolamide (250 mg/day)	3	- No benefit in 2 of 3 - Of limited structural and functional benefit in 1 of 3		Not reported
Bhavsar et al. (2015)	Topical dorzolamide	1	Central foveal thickness decreased from 289 to 258 μm (complete resolution) in one eye and from 456 to 325 in the other (partial resolution)	Not reported	Not reported
Hong et al. (2016)	Oral acetazolamide	1	Complete resolution of CMO	20/50 to 20/25	None
Parikh et al. (2016)	- Cessation of HCQ in 1 - Systemic immunosuppression (e.g., steroids, mycophenolate mofetil) and intravitreal triamcinolone in 1	2	- Resolved after drug cessation - No response	- Not reported - 20/30 in the right eye and 20/25 in the left eye	Not reported
Kim et al. (2018)	Topical dorzolamide	2	Complete resolution of CMO	20/50 OD and 20/40 OS to 20/25 OU in one case, 20/30 OU to 20/25 OD and 20/20 OS in the other	None

Dexamethasone implants have shown promising results for the treatment of CMO in other retinal degenerative diseases such as retinitis pigmentosa. [5] In CMO combined with RP, the therapy resulted in complete resolution of CMO, whereas other treatments such as anti-vascular endothelial growth factor therapy resulted in only partial resolution of macular oedema. [5] Our results, together with those in the literature, suggest that dexamethasone implant therapy is very effective for resolution of macular oedema in eyes with HCQ retinopathy. Although the mechanism of CMO in HCQ retinopathy is unclear, impairment of the blood-retinal barrier (BRB) might be a potential pathogenic mechanism, and the stabilization of the BRB mediated by steroids may have led to resolution of CMO in our case. However, absence of baseline FFA and thus no evidence of fluorescein leakage at baseline in our case makes it impossible to draw a conclusion that the CMO was associated with disruption of the BRB. Further studies should be performed to elucidate the pathogenic mechanism of CMO and the effect of steroid therapy on CMO in HCQ retinopathy. Although our case strongly suggests the CMO-resolving effect of dexamethasone implant, spontaneous resorption of CMO, which can be asymmetric between the treated and untreated eyes, is also possible.

In conclusion, our case suggests that an intravitreal dexamethasone implant can be an effective therapeutic alternative for CMO associated with HCQ retinopathy. Future studies with a larger number of patients and longer follow-up periods are required to assess the efficacy and safety of the treatment.

## Abbreviations

BCVA: Best corrected visual acuity
BRB: Blood-retinal barrier
CFT: Central foveal thickness
CMO: Cystoid macular oedema
FA: Fluorescein angiography
FAF: Fundus autofluorescence
HCQ: Hydroxychloroquine
OCT: Optical coherence tomography
SLE: Systemic lupus erythematosus
VF: Visual field
